# Multilocus Genotyping Reveals New Molecular Markers for Differentiating Distinct Genetic Lineages among “*Candidatus* Phytoplasma Solani” Strains Associated with Grapevine Bois Noir

**DOI:** 10.3390/pathogens9110970

**Published:** 2020-11-21

**Authors:** Alessandro Passera, Yan Zhao, Sergio Murolo, Roberto Pierro, Emilija Arsov, Nicola Mori, Abdelhameed Moussa, Maria R. Silletti, Paola Casati, Alessandra Panattoni, Wei Wei, Sasa Mitrev, Alberto Materazzi, Andrea Luvisi, Gianfranco Romanazzi, Piero A. Bianco, Robert E. Davis, Fabio Quaglino

**Affiliations:** 1 Department of Agricultural and Environmental Sciences, Production, Landscape, Agroenergy, University of Milan, 20133 Milano, Italy; alessandro.passera@unimi.it (A.P.); abdelhameed.moussa@unimi.it (A.M.); paola.casati@unimi.it (P.C.); piero.bianco@unimi.it (P.A.B.); 2 Molecular Plant Pathology Laboratory, USDA-Agriculture Research Service, Beltsville, MD 20705, USA; yan.zhao@usda.gov (Y.Z.); wei.wei@usda.gov (W.W.); robert.davis@usda.gov (R.E.D.); 3 Department of Agricultural, Food and Environmental Sciences, Marche Polytechnic University, 60131 Ancona, Italy; s.murolo@univpm.it (S.M.); g.romanazzi@univpm.it (G.R.); 4 Department of Agriculture, Food and Environment, University of Pisa, 56124 Pisa, Italy; rob.pierro@outlook.it (R.P.); alessandra.panattoni@unipi.it (A.P.); alberto.materazzi@unipi.it (A.M.); 5 Department for Plant and Environment Protection, Faculty of Agriculture, Goce Delcev University, Štip 2000, North Macedonia; emilija.arsov@ugd.edu.mk (E.A.); sasa.mitrev@ugd.edu.mk (S.M.); 6 Department of Biotechnology, University of Verona, 37134 Verona, Italy; nicola.mori@univr.it; 7 National Research Centre, Pests and Plant Protection Department, Agricultural & Biological Research Division, Giza 12622, Egypt; 8 Centro di Ricerca e Sperimentazione in Agricoltura Basile Caramia, 70010 Locorotondo, Italy; mariarsilletti@crsfa.it; 9 Department of Biological and Environmental Sciences and Technologies, University of Salento, 73100 Lecce, Italy; andrea.luvisi@unisalento.it; 10 Institute for Sustainable Plant Protection, National Research Council (IPSP-CNR), Strada delle Cacce 73, 10135 Turin, Italy

**Keywords:** elongation Factor-Tu, hemolysin, restriction fragment length polymorphism (RFLP), *Vitis vinifera* L.

## Abstract

Grapevine Bois noir (BN) is associated with infection by “*Candidatus* Phytoplasma solani” (CaPsol). In this study, an array of CaPsol strains was identified from 142 symptomatic grapevines in vineyards of northern, central, and southern Italy and North Macedonia. Molecular typing of the CaPsol strains was carried out by analysis of genes encoding 16S rRNA and translation elongation factor EF-Tu, as well as eight other previously uncharacterized genomic fragments. Strains of *tuf*-type a and b were found to be differentially distributed in the examined geographic regions in correlation with the prevalence of nettle and bindweed. Two sequence variants were identified in each of the four genomic segments harboring *hlyC*, *cbiQ*-*glyA*, *trxA*-*truB*-*rsuA*, and *rplS*-*tyrS*-*csdB*, respectively. Fifteen CaPsol lineages were identified based on distinct combinations of sequence variations within these genetic loci. Each CaPsol lineage exhibited a unique collective restriction fragment length polymorphism (RFLP) pattern and differed from each other in geographic distribution, probably in relation to the diverse ecological complexity of vineyards and their surroundings. This RFLP-based typing method could be a useful tool for investigating the ecology of CaPsol and the epidemiology of its associated diseases. Phylogenetic analyses highlighted that the sequence variants of the gene *hlyC*, which encodes a hemolysin III-like protein, separated into two clusters consistent with the separation of two distinct lineages on the basis of *tufB* gene sequences. Alignments of deduced full protein sequences of elongation factor-Tu (*tufB* gene) and hemolysin III-like protein (*hlyC* gene) revealed the presence of critical amino acid substitutions distinguishing CaPsol strains of *tuf*-type a and b. Findings from the present study provide new insights into the genetic diversity and ecology of CaPsol populations in vineyards.

## 1. Introduction

Bois noir (BN), a grapevine disease associated with “*Candidatus* Phytoplasma solani” (CaPsol) infection, causes typical grapevine yellows (GY) symptoms and results in important crop losses in the majority of vine-growing European countries, in the Middle East, and in South America [1,2]. Due to the involvement of multiple insect vectors and plant hosts, the biological cycle of CaPsol is extremely complex [3,4,5,6,7,8,9,10], hindering the development of control strategies for effective control of BN epidemics [11]. Molecular markers of genetic diversity among grapevine-affecting phytoplasmas set a solid foundation to improve knowledge of BN epidemiology. In Europe, sequence analysis of translation elongation factor EF-Tu gene *tufB* revealed two main *tuf*-types of CaPsol (*tuf*-type a and *tuf*-type b) present in diseased grapevines, as well as in alternative plant hosts nearby [3], suggesting that ecological differences could be associated with molecular diversification of CaPsol populations and their differential distributions. Numerous studies on molecular typing, based on anaylses of nucleotide sequences of more variable genes (e.g., *secY*, *stamp*, *vmp1*), showed large variability among CaPsol strain populations, shedding light on differences in virulence, origin, and host range of different strains [10,12,13,14,15]. Multiple gene typing analysis was applied to investigate genetic diversity in various bacterial taxa [16,17,18,19]. Such molecular typing was also used to improve knowledge in phytoplasma classification [1,20,21,22,23] and improve knowledge of the epidemiology of phytoplasmal diseases [4,6,7,8,24]. In the present study, molecular characterization of CaPsol phytoplasma strains from Italian and North Macedonian vineyards was carried out by analyses of 16S rRNA and *tufB* genes and eight other previously uncharacterized genomic fragments. The study identified new molecular markers useful for fine differentiation of CaPsol genetic lineages associated with different biological and geographic features.

## 2. Results and Discussion

### 2.1. CaPsol Identification

**Figure 1:**
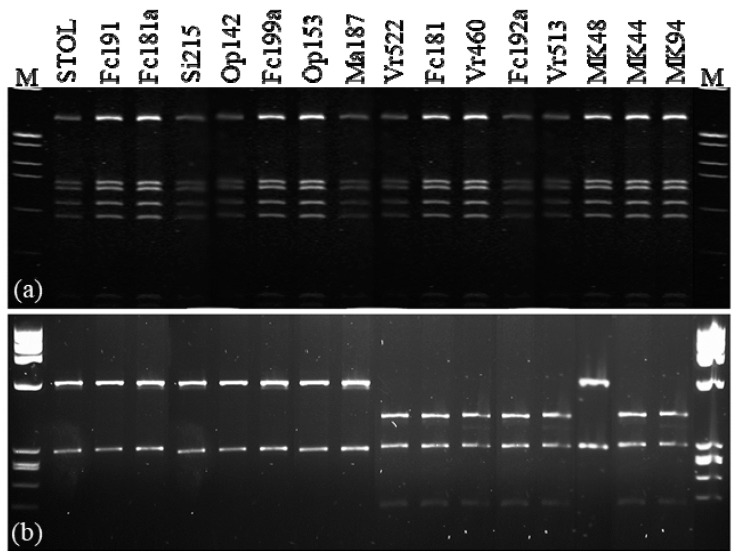
Restriction profiles obtained from the enzyme digestions of nested PCR amplicons of (**a**) 16S rRNA gene with the enzyme *Mse*I, and (**b**) *tufB* gene with the enzyme *Hpa*II.

The primer pair R16F1/R16R1, which is known to prime amplification of 16S rDNA from phytoplasmas classified in groups 16SrI and 16SrXII by PCR [25], was used to characterize 142 DNA samples from grapevines. All symptomatic samples yielded amplicons of approximately 1.1 kb. As expected, positive-control PCRs containing template DNA derived from periwinkle plants infected by the phytoplasma reference strain STOL also produced an amplicon of the same size, whereas negative-control PCRs containing healthy periwinkle DNA or water instead of DNA showed no observable DNA amplification following electrophoresis on agarose gels. All 142 amplicons from symptomatic field samples yielded *Mse*I-RFLP patterns, visualized by electrophoresis on agarose gels, that were indistinguishable from one another and from the pattern typical of the reference strain STOL, indicating that the strains detected in diseased grapevines and other hosts were members of the subgroup 16SrXII-A. The *Mse*I restriction patterns of 15 representative samples are shown in Figure 1a.

### 2.2. Characterization and Distribution of CaPsol tuf-Types

Fragments of *tufB* genes were amplified from all the grapevine samples in nested PCRs using the primer pair fTufAY/rTufAY. Two different *Hpa*II-RFLP patterns were found among digested amplicons (Figure 1b). These two patterns were identical to those previously reported for CaPsol *tuf*-type a and *tuf*-type b, respectively [3]. These results were consistent with previous findings of two *tuf*-types present in vineyards of northern [5,8], central [10,12], and southern [26] Italy. In the present work, CaPsol *tuf*-type a and *tuf*-type b were detected in 49.3% and 50.7% of the 142 symptomatic grapevine samples tested, respectively, but the two CaPsol *tuf*-types were differentially distributed (Table 1). In northern Italy, 63 out of 85 (74.1%) symptomatic vines carried CaPsol *tuf*-type a, and in southern Italy, 30 out of 32 (91.7%) symptomatic vines carried CaPsol *tuf*-type b. In central Italy, the prevalent CaPsol population was also *tuf*-type b, as 14 out of 16 symptomatic vines carried CaPsol strains of this lineage. It is worth noting that central Italy has a similar latitude to North Macedonia. A previous study revealed that the dominant CaPsol type in North Macedonia was also *tuf*-type b [27]. Such differential distributions of CaPsol *tuf*-type a and *tuf*-type b in northern and central/southern Italy is conceivably linked to ecological differences, particularly the potential CaPsol reservoirs in the respective regions. The polyphagous planthopper *Hyalesthes obsoletus* is a known vector of CaPsol. It was reported that nettle (*Urtica dioica* L.) is the main host plant of *H. obsoletus* in northern Italy [28], while bindweed (*Convolvulus arvensis* L.) is a major host of the planthopper in central and southern Italy [26]. Since nettle and bindweed are likely reservoirs of CaPsol inoculum, it would be interesting to learn whether they also play a role as niches for the differentiation and adaptation of these two distinct CaPsol types.

In the present study, a close examination of the CaPsol populations within Northern Italy unveiled a striking difference in distribution of the two CaPsol *tuf*-types in vineyards of Lombardy vs Veneto regions: while there was a high prevalence of *tuf*-type a (92.3%) in Veneto, an almost even distribution of CaPsol *tuf*-type a (58.7%) and *tuf*-type b (41.3%) was found in Lombardy. These differential distribution patterns within northern Italy could be explained by the differences in weeds present within the vineyards. In fact, in Europe, CaPsol *tuf*-type a and *tuf*-type b are mainly associated with nettle and bindweed, respectively [3]. Previous studies indicated that nettle is highly present within vineyards in Veneto [5], while in Lombardy it is found mainly in the surroundings rather than in the vineyards [8]. Likewise, in central and southern Italy where rainfall is limited in the summer, nettle is less frequent and bindweed prevails [29]. These considerations reinforce the idea that, even within a given geographic area, the variation in the prevalence of weed species among vineyards influences the composition of CaPsol strain populations in nearby grapevine plants [15].

### 2.3. Possible Role of Protein Encoded by tufB Gene (EF-Tu) in Host Selection

**Figure 2:**
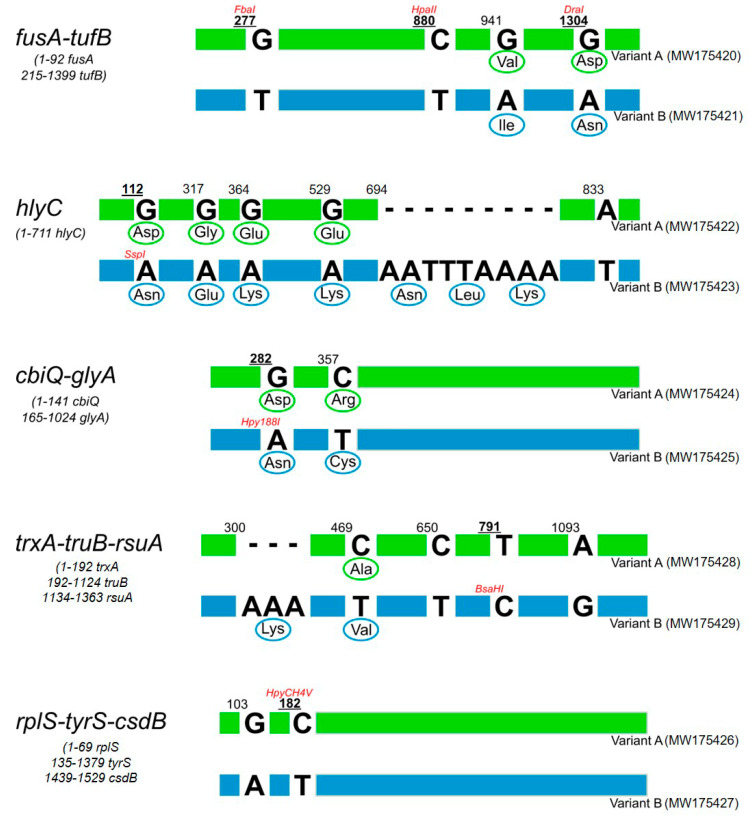
Single nucleotide polymorphism (SNP) positions, predicted enzymatic restriction sites, and amino acid substitutions distinguish sequence variance in each CaPsol genomic fragment, *fusA-tufB*, *hlyC, cbiQ-glyA, trxA-truB-rsuA,* and *rplS-tyrS-csdB***.**

Forty symptomatic vine samples, representing seven geographic regions, were selected for further analyses. Half of the vine samples were infected with *tuf*-type a CaPsol strains and the other half were infected with *tuf*-type b CaPsol strains (Table 2). Nested PCR conducted with primer pairs fusAF2/tufBR1 allowed the amplification of a 1399 bp DNA segment from the 40 samples. The nucleotide sequences of the amplicons were determined, with each amplicon containing a partial *fusA* gene (1–92 bp) and a full-length *tufB* gene (215–1399 bp). Alignment of the sequences confirmed the presence of two *tufB* sequence variants characteristic of *tuf*-type a (Acc. No. MW175420) and b (Acc. No. MW175421), respectively, distinguished on the basis of four single nucleotide polymorphisms (SNPs), positioned in the *tufB* gene at nucleotides 277, 880, 941, and 1304 relative to the annealing site of the primer fusAF2 (Figure 2). The SNP at nucleotide 880 (C/T) differentiated between *tuf*-type a and b; this SNP accounts for the difference in *Hpa*II-RFLP patterns, as previously reported [3] (Figure 1b and Figure 2). Full protein sequences (344 amino acids) of elongation factor Tu obtained from *in silico* translation of *tufB* nucleotide sequences related to *tuf*-type a and b were aligned. The alignment revealed differences in amino acid composition at positions 243 (Val/Ile, corresponding to an SNP at nucleotide position 941) and 364 (Asp/Asn, corresponding to an SNP at nucleotide position 1304) (Figure 2). Previous studies on plants and humans indicated that a single substitution between Val and Ile or between Asp and Asn could modify receptor binding activity [30] or enzymatic catalytic activity [31,32]. Thus, key amino acid substitutions in CaPsol *tufB* genes could also change EF-Tu activity and/or modify interactions with its binding protein(s). Although EF-Tu is well known as a cytoplasmic protein involved in translation [33], it was reported that EF-Tu can (i) be localized at the cell surface and act as a virulence factor [34], and (ii) interact with virulence factors inside the bacterial cytoplasm [35]. Previous studies reported differences in EF-Tu protein sequences of CaPsol strains infecting grapevine in Austria and Iran [36,37], and suggested that these differences in EF-Tu primary structure may act as CaPsol fitness factors in host selection.

### 2.4. Genetic Lineages in CaPsol Strains Determined by Multiple Gene Sequence Typing

Nested PCR reactions, using different specific primer pairs (Table 3), allowed amplification of the eight genomic fragments (*cbiQ*-*glyA*, *rplS*-*tyrS*-*csdB*, *trxA*-*truB*-*rsuA*, *hlyC*, *potC*-*potD*, *pnp*, *gyrA*-*gyrB*, *aspS*-*mesJ*) from all 40 selected vines (Table 2). The analyzed genes encode proteins involved in different cell functions, including: (i) replication, transcription, and translation processes (*aspS*, encoding an aspartyl-tRNA synthetase; *csdB*, encoding nifS-like protein; *gyrA*, encoding DNA gyrase subunit A, *gyrB*, encoding DNA gyrase subunit B; *glyA*, encoding glycine hydroxymethyltransferase; *mesJ*, encoding tRNA(Ile)-lysidine/2-thiocytidine synthetase; *pnp*, encoding polyribonucleotide nucleotidyltransferase; *rplS*, encoding 50S ribosomal protein L19; *rsuA*, encoding 16S rRNA pseudouridylate synthase; t*yrS*, encoding tyrosyl-tRNA synthetase; and *truB*, encoding tRNA pseudouridine synthase B); (ii) antioxidant activities (*trxA*, encoding thioredoxin); (iii) transport systems (*cbiQ*, encoding an ABC-type cobalt transport protein CbiQ; *potC*, encoding a spermidine/putrescine ABC transporter, permease protein; *potD*, encoding spermidine/putrescine ABC transporter, periplasmic protein); and (iv) toxicity (*hlyC*, encoding hemolysin III-like protein). Obtained amplicons were sequenced and analyzed. Nucleotide sequence alignments revealed that (i) two sequence variants were identified in each of four genomic fragments (*hlyC*, *cbiQ*-*glyA*, *trxA*-*truB*-*rsuA*, and *rplS*-*tyrS*-*csdB*), and (ii) no sequence variation was identified in the remaining genomic fragments (*potC*-*potD*, *pnp*, *gyrA*-*gyrB*, and *aspS*-*mesJ*). One representative sequence for each of these four fragments was deposited into NCBI GenBank under Acc. No. MW175430-MW175433.

Figure 2 shows the SNP position, differential restriction sites, and the corresponding amino acid variations in deduced protein sequences for each sequence variant. In the genomic fragment *hlyC*, the two sequence variants *hlyC*-A (Acc. No. MW175422) and -B (Acc. No. MW175423) were distinguished by five SNPs and a nine-nucleotide indel, differentiating the predicted protein sequences by seven amino acid substitutions. In the genomic fragment *cbiQ*-*glyA*, the two sequence variants *cbiQ*-*glyA*-A (Acc. No. MW175424) and -B (Acc. No. MW175425) were distinguished by two nonsynonymous SNPs. In the genomic fragment *trxA*-*truB*-*rsuA*, the two sequence variants *trxA*-*truB*-*rsuA*-A (Acc. No. MW175428) and -B (Acc. No. MW175429) were distinguished by four SNPs and a three-nucleotide indel, differentiating the predicted protein sequences by two amino acid substitutions. In the genomic fragment *rplS*-*tyrS*-*csdB*, the two sequence variants *rplS*-*tyrS*-*csdB*-A (Acc. No. MW175426) and -B (Acc. No. MW175427) were distinguished by two synonymous SNPs.

**Figure 3:**
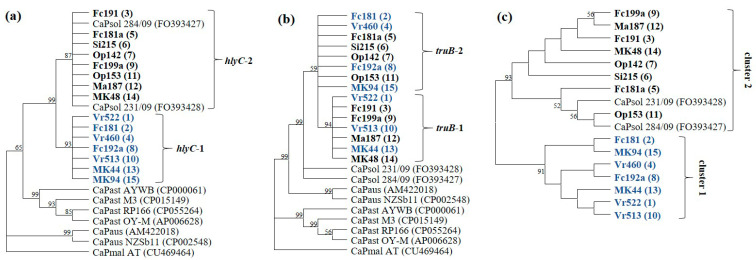
Unrooted phylogenetic trees constructed based on the nucleotide sequence alignment of *hlyC* (**a**) and *truB* (**b**) genes, and the *hlyC*, *glyA*, *truB*, and *tyrS* concatenated gene sequences (**c**). The highest log likelihood values of trees a, b, and c are −3001.71, −3427.33, and −5020.67, respectively. The percentages of trees in which the associated taxa clustered together are shown next to the branches. This analysis involved 24 sequences with a total of 748 positions (a), 24 sequences with a total of 892 positions (b), and 17 sequences with a total of 3778 positions (c). Strains representing the CaPsol lineages identified in this study are in bold. The number (1–15) in parentheses represents the CaPsol strain’s lineage; CaPsol strains clustered into *tuf*-type a and b are indicated in blue and black, respectively.

From the CaPsol strains identified in the 40 selected vines, 15 multiple gene profiles associated with distinct CaPsol lineages (named as CaPsol lineage 1 to 15) were determined by the combination of genomic fragment sequence variants (Table 2). The result from a phylogenetic analysis of *hlyC* gene sequences demonstrated that the two *hlyC* sequence variants grouped into two clusters (*hlyC*-1 and -2) were consistent with those identified on the basis of *tufB* gene sequences (Figure 3a and Figure S1a). In phytoplasmas, *hlyC* gene encodes a hemolysin III-like protein. It was reported that in humans [38] and other plant pathogens [39], hemolysins act as virulence factors. While it remains unknown whether hemolysin III-like proteins are involved in phytoplasma virulence and/or fitness in different hosts, our finding that the separation of the two CaPsol *hlyC* gene sequence variants (lineages) was parallel to the separation of the two CaPsol *tuf* types delineated previously certainly raises this possibility.

Two distinct clusters of sequence variants were also identified based on the alignment of the *truB*, *glyA,* and *tyrS* genes. While the sequence alignment alone did not provide a clear picture as to whether such clustering was parallel to the separation of the two CaPsol *tuf* types, results from a phylogenetic analysis on the concatenated *hlyC*, *truB*, *glyA*, and *tyrS* gene sequences showed that the 15 CaPsol lineages were grouped into two distinct clusters (cluster-1 and -2) (Figure 3b and Figure S1a–c), consistent with the separation of the two lineages delineated on the basis of *tufB* genes (Figure 3c). Further studies should be conducted to investigate whether EF-Tu and/or hemolysin III-like protein are involved in interactions of CaPsol with host plants and/or insect vectors, thereby driving adaptation to varied vineyard ecosystems.

### 2.5. RFLP Analyses: Prevalence of CaPsol Lineages

**Figure 4:**
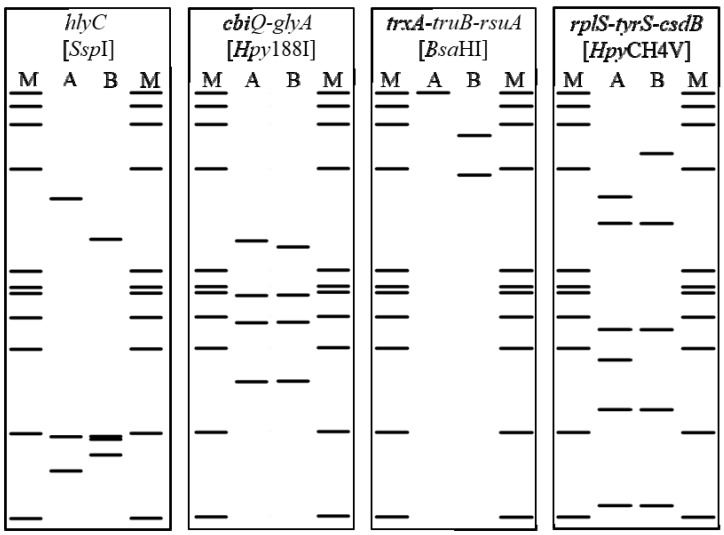
*In silico* restriction profiles reveal sequence variance of CaPsol strains by analyzing the genomic fragments *hlyC*, *cbiQ-glyA*, *trxA-truB-rsuA*, and *rplS*, *tyrS-csdB*.

*In silico* digestion, carried out on the representative nucleotide sequences of CaPsol sequence variants of the *hlyC*, *cbiQ*-*glyA*, *trxA*-*truB*-*rsuA*, and *rplS*-*tyrS*-*csdB* genomic fragments, allowed generation of virtual RFLP profiles for the restriction enzymes *Ssp*I, *Hpy*188I, *Bsa*HI, and *Hpy*CH4V, respectively (Figure 4). For each genetic locus analyzed, the obtained virtual RFLP profiles consistently differentiated the two sequence variants identified in the present study (Figure 2). In vitro RFLP assays, conducted on nested PCR products amplified from the 40 selected vines, produced the predicted restriction profiles for each sequence variant of each genomic fragment (data not shown). Having determined the resolution power of these RFLP assays, the CaPsol strains identified in the remaining 102 vines were attributed to lineages using in vitro digestion (Table 1). Based on the obtained collective RFLP patterns, each CaPsol strain was attributed to one of the 15 previously determined lineages (Table 1). Molecular markers distinguishing such lineages can be exploited to study different aspects of BN disease, such as CaPsol strain population structure determination and epidemiology in different agroecosystems. In particular, the major contribution to the variability among these lineages is due to SNPs within the *cbiQ*-*glyA*, *trxA*-*truB*-*rsuA*, and *rplS*-*tyrS*-*csdB* genomic fragments. In fact, numerous studies reported the usefulness of genes not directly related to phytoplasma virulence (e.g., *rplV*-*rpsC*, *groEL*, *map*) as valuable markers for phytoplasma strain typing related to their ecology [40,41,42].

The prevalence of CaPsol lineages was evaluated in the different geographic areas under study, leading to new insights into CaPsol populations in Italinan vineyards: (i) three lineages (13, 14, 15) were found exclusively in North Macedonia and not in Italy; (ii) a single lineage (6) was found in southern Italy; (iii) four lineages were found in central Italy, with two in Marche (10, 12) and two in Tuscany (5, 11); (iv) six lineages (1, 2, 4, 6, 7, 10) were found in Veneto, three of which (1, 4, 10) represented 87% of CaPsol strains; (v) twelve lineages (1 to 12) were found in Lombardy, three of which (1, 4, 7) represented 65% of CaPsol strains, with three (3, 8, 9) found exclusively in this region (Table 1). Notably, a low number of CaPsol lineages, reflecting low genetic diversity within strain populations, was found in the geographic areas (Veneto, Marche, Tuscany, Apulia, Sicily, North Macedonia) where high prevalence of a single *tuf*-type was reported. In contrast, numerous CaPsol lineages, reflecting an elevated genetic diversity within strain populations, were found in Lombardy where the two *tuf*-types (a and b) were equally present. Such genetic heterogeneity within the CaPsol population in Lombardy was also noted in a recent study based on CaPsol molecular characterization using the hypervariable gene *stamp* [8]. It is reasonable to hypothesize that this variability in CaPsol strains could be related to the ecological complexity of vineyards and their surroundings, including the presence of multiple insect vectors and alternative plant hosts [8]. The RFLP-based typing method used in the present study could be considered to be a valuable tool for research on the ecology of CaPsol and the epidemiology of its associated diseases.

## 3. Materials and Methods

### 3.1. Sample Collection

From 2015 to 2018, leaf samples were collected from 142 symptomatic grapevine plants in BN-affected vineyards in Lombardy and Veneto regions (northern Italy), Marche, and Tuscany regions (central Italy), Apulia and Sicily regions (southern Italy), and the Republic of North Macedonia (Table 1). For each plant sample, 1 g of leaf petioles was stored at −30 °C until molecular analysis.

### 3.2. CaPsol Molecular Identification

Total nucleic acids were extracted from the leaf petioles of the examined plants, as previously described [43]. Detection and identification of CaPsol were carried out by means of nested PCR amplification of 16S rDNA primed by the universal primer pairs P1/P7 [44] and followed by the 16SrI group-specific primer pair R16F1/R16R1 [25], with a subsequent *Mse*I-RFLP assay performed on the obtained amplicons. PCR and RFLP reaction conditions were as previously described [45]. PCRs were performed using Taq polymerase (Promega, Milan, Italy) in an automated thermal cycler (MasterCycler Gradient, Eppendorf, Milan, Italy). PCR and enzymatic digestion products were electrophoresed through 1% and 3% agarose gel, respectively, in Tris-Borate- Ethylenediaminetetraacetic acid (TBE) buffer, stained with Midori Green Advance (Biosigma, Venice, Italy), and visualized under a UV transilluminator. Total nucleic acids from periwinkle (*Catharanthus roseus* (L.) G. Don) infected by the phytoplasma strain STOL (CaPsol, subgroup 16SrXII-A) was used as the reference control. Total nucleic acids extracted from the healthy periwinkle and PCR mixture devoid of nucleic acids was used as the negative control.

### 3.3. Molecular Characterization of CaPsol Strains through Multilocus Genotyping Analysis

The *tufB* genotyping of CaPsol strains identified in infected samples was performed by nested PCR amplification using the primer pair fTuf1/rTuf1 followed by fTufAY/rTufAY, with subsequent *Hpa*II-RFLP assays performed on the obtained amplicons [3]. The experimental controls, PCR conditions, and PCR-RFLP analysis were same as the above 16S rRNA gene analysis.

Multilocus genotyping analysis was carried out by employing 40 CaPsol strains representing distinct *tuf*-types (20 *tuf*-type a and 20 *tuf*-type b) and different geographic origins. Based on the genome sequences of CaPsol strain 284/09 (FO393427) and 231/09 (FO393428) [46], primer pairs were designed to amplify the complete *tufB* gene sequence and 8 genomic fragments (*cbiQ*-*glyA*, *rplS*-*tyrS*-*csdB*, *trxA*-*truB*-*rsuA*, *hlyC*, *potC*-*potD*, *pnp*, *gyrA*-*gyrB*, *aspS*-*mesJ*) containing 16 previously uncharacterized genes encoding proteins (Table 3). The experimental controls, PCR conditions, and PCR-RFLP analysis were same as the above 16S rRNA gene analysis. Obtained amplicons of the full *tufB* gene and the 8 genomic fragments were sequenced in both senses (5X coverage per base position) by a commercial service (Eurofins Genomics, Germany). Nucleotide sequences were compiled in FASTA format, assembled by employing the Contig Assembling Program of the software BioEdit version 7.2 [47], and trimmed to the annealing sites of the related primers utilized by the nested PCRs. For each genomic fragment under study, nucleotide sequences were aligned using the ClustalW Multiple Alignment application within the software BioEdit, and single nucleotide polymorphisms (SNPs), restriction enzymatic sites, and deduced amino acid substitution were searched for.

### 3.4. Phylogenetic Analyses

Phylogenetic trees were established by aligning the nucleotide sequences of *tufB*, *hlyC*, *glyA*, *truB*, and *tyrS* genes of CaPsol strains representing genetic lineages identified in this study and those of available phytoplasma strains in National Center for Biotechnology Information (NCBI) GenBank. Moreover, phylogenetic analysis was also performed on *hlyC*, *glyA*, *truB*, and *tyrS* concatenated nucleotide sequences. The evolutionary history was inferred using the Maximum Likelihood method and the Tamura–Nei model [48]. Initial trees for the heuristic search were obtained automatically by applying Neighbor-Join and BioNJ algorithms to a matrix of pairwise distances estimated using the Maximum Composite Likelihood (MCL) approach, and then selecting the topology with the superior log likelihood value. Evolutionary analyses were conducted in MEGA X [49].

### 3.5. Survey on CaPsol Genetic Lineages by Restriction Fragment Length Polymorphism Analyis

SNPs distinguishing CaPsol genetic lineages were checked for their position in recognition sites for restriction enzymes by *in silico* restriction fragment length polymorphism (RFLP) assays using the software pDRAW32 (AcaClone Software, http://acaclone.com). The obtained virtual RFLP profiles were confirmed by actual digestion of genomic fragments amplified from the 40 grapevines selected for multiple gene sequencing. Nested PCR amplification of the genomic fragments (*hlyC*, *cbiQ*-*glyA*, *trxA*-*truB*-*rsuA*, and *rplS*-*tyrS*-*csdB*) was conducted on the remaining 102 CaPsol-infected grapevines (not included in the multilocus genotyping). Actual RFLP analyses were performed using the enzyme *Ssp*I on *hlyC* amplicons, *Hpy*188I on *cbiQ*-*glyA* amplicons, *Bsa*HI on *trxA*-*truB*-*rsuA* amplicons, and *Hpy*CH4V on *rplS*-*tyrS*-*csdB* amplicons, respectively. Digestion reactions were carried out as indicated by the enzyme manufacturer’s instructions (New England Biolabs, Ipswich, MA, USA). RFLP profiles were visualized by electrophoresis on 3% agarose gel.

## Figures and Tables

**Table 1:** Prevalence of *tufB* types and CaPsol lineages in considered geographic areas.

Country	Region	No. of Samples	*tuf-*Type a	*tuf-*Type b	No. of CaPsol Strains in Each Lineage														
					1	2	3	4	5	6	7	8	9	10	11	12	13	14	15
Italy (North)	Lombardy	46	27	19	11	4	1	9	3	2	10	1	1	2	1	1			
	Veneto	39	36	3	13	2		11		1	2			10					
Italy (Center)	Marche	16	2	14										2		14			
	Tuscany	16	0	16					6						10				
Italy (South)	Apulia	6	0	6						6									
	Sicily	3	0	3						3									
Macedonia (North)		16	2	14					2	6	3				1		1	2	1
	Overall	142	67	75	24	6	1	20	11	18	15	1	1	14	12	15	1	2	1

**Table 2:** Fifteen *Ca*Psol lineages determined by collective sequence variance/RFLP profiles.

Origin	*Ca*Psol Strain	Sequence Variant/RFLP Profile					*Ca*Psol
		*fusA-tufB*	*hlyC*	*cbiQ-glyA*	*trxA-truB-rsuA*	*rplS-tyrS-csdB*	Lineage
Lombardy	Fc180	A (*tuf*-type a)	A	A	A	A	1
(North Italy)	Fc181	A	A	A	B	A	2
	Fc182	A	A	A	A	A	1
	Fc183	A	A	A	B	A	2
	Fc185	A	A	A	A	A	1
	Fc191	B (*tuf*-type b)	B	A	A	B	3
	Fc181a	B	B	B	B	A	5
	Fc192a	A	A	B	B	B	8
	Fc199a	B	B	B	A	B	9
	Op142	B	B	A	B	B	7
	Op153	B	B	B	B	B	11
	Op280	A	A	B	B	A	4
	Op282	A	A	B	B	A	4
	Op304	A	A	B	B	A	4
Veneto	Vr460	A	A	B	B	A	4
(North Italy)	Vr462	B	B	A	B	A	6
	Vr501	B	B	A	B	B	7
	Vr509	B	B	A	B	B	7
	Vr513	A	A	B	A	A	10
	Vr517	A	A	B	A	A	10
	Vr522	A	A	A	A	A	1
Marche	Ma187	B	B	B	A	A	12
(Center Italy)	Ma189	B	B	B	A	A	12
	Ma190	A	A	B	A	A	10
	Ma191	A	A	B	A	A	10
Tuscany	San11/15	B	B	B	B	B	11
(Center Italy)	San15/15	B	B	B	B	B	11
	San17/15	B	B	B	B	B	11
	San5/16	B	B	B	B	B	11
Apulia	Pu123	B	B	A	B	A	6
(South Italy)	Pu124	B	B	A	B	A	6
	Pu125	B	B	A	B	A	6
Sicily	Si212	B	B	A	B	A	6
(South Italy)	Si214	B	B	A	B	A	6
	Si215	B	B	A	B	A	6
North	MK44	A	A	A	A	B	13
Macedonia	MK48	B	B	A	A	A	14
	MK61	B	B	A	B	A	6
	MK62	B	B	A	B	A	6
	MK94	A	A	A	B	B	15

**Table 3:** Primer sequences designed for molecular typing and expected PCR product sizes.

Gene	Primer	Sequence (5’–3’)	PCR	Nested PCR
				Product Size (nt)
*fusA-tufB*	fusAF1	CTTTCTGARATGTTTGGMTATGCTAC	d	1399
	fusAF2	GCGTTCCAATACYCAAGGAAGAG	n	
	tufBR1	ACAAAGCTCCAACGTTATCGCCTGC	d/n	
*cbiQ-glyA*	cbiQF1	AGAGGTTATGTATTGGGAGCG	d/n	1024
	glyAR1	CAAAGAACTTGCAAGAGTTTGGGC	d	
	glyAR2	TGTTGATAATCTTTAAAGGCAGG	n	
*rplS-tyrS-csdB*	rplSF1	CCTGTGCACTCCCCTAATAACGA	d	1529
	csdBR1	ACCTTCTTGGAGTGTTTCGCCTAGAC	d	
	rplSF2	CGTCGTGCTAAGTCGCATTACG	n	
	csdBR2	GTTTCAAAGAGGTAGCCGCATTATCG	n	
*trxA-truB-rsuA*	trxAF1	TGCCAATTGGTGTGGTCCATGTC	d	1363
	truBR3	GCCTCTATGATCAAATCAAGGACAG	d	
	trxAF2	GAATTATCACAATCAGAACAGGGTG	n	
	truBR4	TCTTTGGCGGTCGAAAGGTAGCC	n	
*hlyC*	hlysF1	ATKATTVTGAAATGKBCTAC	d	914
	ackAR2	GAAATTTTAAAGAAGARCTAC	d	
	hlysF2	ATKATTVTGAAATGKBCTACYAAAMMAAC	n	
	ackAR3	AGAAGARCTACCWGAATTWACCGAC	n	
*potD*	potF1	ACGATTAATCCAACTGTTAATGC	d	953
	potR1	TACTTGGATAAGCAATGATGTC	d	
	potF2	AGGGTGAATATTTAGACCCTCAAAC	n	
	potR2	TGGATAAGCAATGATGTCATTCC	n	
*pnp*	pnpF1	TGCTAGAAATGTGGATGCTTCTG	d	1448
	pnpR1	TGACATTTCTTGGCGTGGAGTG	d	
	pnpF2	GATACAGTAGTTTTATCGGCTAC	n	
	pnpR2	GTAATACCATCTTTGCTGCCAGC	n	
*gyrA-gyrB*	gyrAF2	TGGGCTTCTTTGATGTCTGCTG	d	1705
	gyrBR2	TGACCGATGCTGACGTTGATGGTG	d	
	gyrAF3	TCTAATTGCAGTATCGATGTC	n	
	gyrBR3	TGCTGACGTTGATGGTGCTCAC	n	
*aspS-mesJ*	aspSF1	GTAGTTGAGATCAAGGGGTTAGTTG	d	1333
	mesJR1	TGAATCAACGCCGCCGCTAACAG	d	
	aspSF2	TCGCAGCCAAGATAGTCTTGAAG	n	
	mesJR2	CTTTTCGACTGTTCCGGGGGAATC	n	

## Supplementary Materials

The following are available online at https://www.mdpi.com/2076-0817/9/11/970/s1, Figure S1: Unrooted phylogenetic trees constructed based on the nucleotide sequence alignment of the genes *tufB* (a), *glyA* (b), and *tyrS* (c).

## References

[1] Quaglino F., Zhao Y., Casati P., Bulgari D., Bianco P.A., Wei W., Davis R.E. (2013). ‘*Candidatus* Phytoplasma solani’, a novel taxon associated with stolbur and Bois Noir related diseases of plants. Int. J. Syst. Evol. Microbiol..

[2] Romanazzi G., Murolo S., Feliziani E. (2013). Effects of an innovative strategy to contain grapevine Bois noir: Field treatment with resistance inducers. Phytopathology.

[3] Langer M., Maixner M. (2004). Molecular characterisation of Grapevine yellows associated phytoplasmas of the stolbur-group based on RFLP-analysis of non-ribosomal DNA. Vitis.

[4] Cvrković T., Jović J., Mitrović M., Krstić Q., Toševski I. (2014). Experimental and molecular evidence of *Reptalus panzeri* as a natural vector of bois noir. Plant Pathol..

[5] Mori N., Quaglino F., Tessari F., Pozzebon A., Bulgari D., Casati P., Bianco P.A. (2015). Investigation on ‘bois noir’ epidemiology in north-eastern Italian vineyards through a multidisciplinary approach. Ann. Appl. Biol..

[6] Kosovac A., Radonjić S., Hrnčić S., Krstić O., Toševski I., Jović J. (2016). Molecular tracing of the transmission routes of bois noir in Mediterranean Vineyards of Montenegro and experimental evidence for the epidemiological role of *Vitex agnus-castus* (Lamiaceae) and associated *Hyalesthes obsoletus* (Cixiidae). Plant Pathol..

[7] Kosovac A., Jakovljević M., Krstić O., Cvrković T., Mitrović M., Toševski I., Jović J. (2019). Role of plant-specialized *Hyalesthes obsoletus* associated with *Convolvulus arvensis* and *Crepis foetida* in the transmission of ‘*Candidatus* Phytoplasma solani’-inflicted Bois noir disease of grapevine in Serbia. Eur. J. Plant Pathol..

[8] Quaglino F., Sanna F., Moussa A., Faccincani M., Passera A., Casati P., Bianco P.A., Mori N. (2019). Identification and ecology of alternative insect vectors of ‘*Candidatus* Phytoplasma solani’ to grapevine. Sci. Rep..

[9] Moussa A., Mori N., Faccincani M., Pavan F., Bianco P.A., Quaglino F. (2019). *Vitex agnus-castus* cannot be used as trap plant for the vector *Hyalesthes obsoletus* to prevent infections by ‘*Candidatus* Phytoplasma solani’ in northern Italian vineyards: Experimental evidence. Ann. Appl. Biol..

[10] Pierro R., Panattoni A., Passera A., Materazzi A., Luvisi A., Loni A., Ginanni M., Lucchi A., Bianco P.A., Quaglino F. (2020). Proposal of a new Bois noir epidemiological pattern related to ‘*Candidatus* Phytoplasma solani’ strains characterized by a possible moderate virulence in Tuscany. Pathogens.

[11] Bianco P.A., Romanazzi G., Mori N., Myrie W., Bertaccini A., Bertaccini A., Weintraub G.P., Rao G.P., Mori N. (2019). Integrated management of phytoplasma diseases. Transmission and Management of Phytoplasma—Associated Diseases. Phytoplasmas: Plant Pathogenic Bacteria–II.

[12] Murolo S., Romanazzi G. (2015). In-vineyard population structure of ‘*Candidatus* Phytoplasma solani’ using multilocus sequence typing analysis. Infect. Gen. Evol..

[13] Quaglino F., Maghradze D., Casati P., Chkhaidze N., Lobjanidze M., Ravasio A., Passera A., Venturini G., Failla O., Bianco P.A. (2016). Identification and characterization of new ‘*Candidatus* Phytoplasma solani’ strains associated with bois noir disease in *Vitis vinifera* L. cultivars showing a range of symptoms severity in Georgia, the Caucasus region. Plant Dis..

[14] Pierro R., Passera A., Panattoni A., Casati P., Luvisi A., Rizzo D., Bianco P.A., Quaglino F., Materazzi A. (2018). Molecular typing of ‘Bois Noir’ phytoplasma strains in the Chianti Classico area (Tuscany, Central Italy) and their association with symptom severity in Vitis vinifera L. cv. Sangiovese. Phytopathology.

[15] Pierro R., Passera A., Panattoni A., Rizzo D., Stefani L., Bartolini L., Casati P., Luvisi A., Quaglino F., Materazzi A. (2018). Prevalence of a ‘*Candidatus* Phytoplasma solani’ strain, so far associated only with other hosts, in Bois Noir-affected grapevines within Tuscan vineyards. Ann. Appl. Biol..

[16] Naser S.M., Vancanneyt M., Hoste B., Snauwaert C., Swings J. (2006). *Lactobacillus cypricasei* Lawson et al. 2001 is a later heterotypic synonym of *Lactobacillus acidipiscis* Tanasupawat et al. 2000. Int. J. Syst. Evol. Microbiol..

[17] Mignard S., Flandrois J.P. (2008). A seven-gene, multilocus, genus-wide approach to the phylogeny of mycobacteria using supertrees. Int. J. Syst. Evol. Microbiol..

[18] Pascual J., Macián M.C., Arahal D.R., Garay E., Pujalte M.J. (2010). Multilocus sequence analysis of the central clade of the genus *Vibrio* by using the 16S rRNA, *recA*, *pyrH*, *rpoD*, *gyrB*, *rctB* and *toxR* genes. Int. J. Syst. Evol. Microbiol..

[19] Adkar-Purushothama C.R., Quaglino F., Casati P., Bianco P.A. (2011). Molecular typing of Coorg black pepper yellows phytoplasma by multiple gene analysis. Ann. Appl. Biol..

[20] Malembic-Maher S., Salar P., Filippin L., Carle P., Angelini E., Foissac X. (2011). Genetic diversity of European phytoplasmas of the 16SrV taxonomic group and proposal of ‘*Candidatus* Phytoplasma rubi’. Int. J. Syst. Evol. Microbiol..

[21] Lee I.M., Bottner-Parker K.D., Zhao Y., Bertaccini A., Davis R.E. (2012). Differentiation and classification of phytoplasmas in the pigeon pea witches’-broom group (16SrIX): An update based on multiple gene sequence analysis. Int. J. Syst. Evol. Microbiol..

[22] Durante G., Casati P., Clair D., Quaglino F., Bulgari D., Boudon-Padieu E., Bianco P.A. (2012). Sequence analyses of S10-spc operon among 16SrV group phytoplasmas: Phylogenetic relationships and identification of discriminating single nucleotide polymorphisms. Ann. Appl. Biol..

[23] Davis R.E., Zhao Y., Dally E.L., Lee I.M., Jomantiene R., Douglas S.M. (2013). ‘*Candidatus* Phytoplasma pruni’, a novel taxon associated with X-disease of stone fruits, *Prunus* spp.: Multilocus characterization based on 16S rRNA, *secY*, and ribosomal protein genes. Int. J. Syst. Evol. Microbiol..

[24] Casati P., Quaglino F., Stern A.R., Tedeschi R., Alma A., Bianco P.A. (2011). Multiple gene analyses reveal extensive genetic diversity among ‘*Candidatus* Phytoplasma mali’ populations. Ann. Appl. Biol..

[25] Lee I.-M., Gundersen D.E., Hammond R.W., Davis R.E. (1994). Use of mycoplasmalike organism (MLO) group-specific oligonucleotide primers for nested-PCR assays to detect mixed-MLO infections in a single host plant. Phytopathology.

[26] Oliveri C., Pacifico D., D’Urso V., La Rosa R., Marzachi C., Tessitori M. (2015). Bois noir phytoplasma variability in a Mediterranean vineyard system: New plant host and putative vectors. Australas. Plant Pathol..

[27] Kostadinovska E., Quaglino F., Mitrev S., Casati P., Bulgari D., Bianco P.A. (2014). Multiple gene analyses identify distinct “bois noir” phytoplasma genotypes in the Republic of Macedonia. Phytopathol. Mediterr..

[28] Mori N., Pavan F., Reggiani N., Bacchiavini M., Mazzon L., Paltrinieri S., Bertaccini A. (2012). Correlation of bois noir disease with nettle and vector abundance in northern Italy vineyards. J. Pest Sci..

[29] Murolo S., Garbarino M., Mancini V., Romanazzi G. (2020). Spatial pattern of Bois noir: Case study of a delicate balance between disease progression and recovery. Sci. Rep..

[30] Berg K.A., Dunlop J., Sanchez T., Silva M., Clarke W.P. (2008). A conservative, single-amino acid substitution in the second cytoplasmic domain of the human serotonin2C receptor alters both ligand-dependent and -independent receptor signalling. J. Pharm. Exp. Therap..

[31] Bruner A.C., Jung S., Abbott A.G., Powell G.L. (2001). The naturally occurring high oleate oil character in some peanut varieties results from reduced oleoyl-PC desaturase activity from mutation of aspartate 150 to asparagine. Crop Sci..

[32] Kawachi T., Sunaga Y., Ebato M., Hatanaka T., Harada H. (2006). Repression of nitrate uptake by replacement of Asp105 by asparagine in AtNTR3.1 in *Arabidopsis thaliana* L.. Plant Cell Physiol..

[33] Kavaliauskas D., Nissen P., Knudsen C.R. (2012). The busiest of all ribosomal assistants: Elongation factor Tu. Biochemistry.

[34] Kunert A., Losse J., Gruszin C., Hiihn M., Kraendler K., Mikkat S., Volke D., Hoffmann R., Jokiranta T.S., Seeberger H. (2007). Immune evasion of the human pathogen *Pseudomonas aeruginosa*: Elongation factor Tuf is a factor H and plasminogen binding protein. J. Immunol..

[35] Archambaud C., Gouin E., Pizarro-Cerda J., Cossart P., Dussurget O. (2005). Translation elongation factor EF-Tu is a target for Stp, a serine-threonine phosphatase involved in virulence of *Listeria monocytogenes*. Mol. Microbiol..

[36] Aryan A., Brader G., Mörtel J., Pastar M., Riedle-Bauer M. (2014). An abundant ‘*Candidatus* Phytoplasma solani’ tuf b strain is associated with grapevine, stinging nettle and *Hyalesthes obsoletus*. Eur. J. Plant Pathol..

[37] Jamshidi E., Murolo S., Salehi M., Romanazzi G. (2020). Sequence analysis of new *tuf* molecular types of ‘*Candidatus* Phytoplasma solani’ in Iranian vineyards. Pathogens.

[38] Goebel W., Chakraborty T., Kreft J. (1998). Bacterial hemolysins as virulence factors. Ant. Van Leeuw..

[39] Gambetta G.A., Matthews M.A., Syvanen M. (2018). The *Xylella fastidosa* RTX operons: Evidence for the evolution of protein mosaics through novel genetic exchanges. BMC Genom..

[40] Quaglino F., Casati P., Bianco P.A. (2010). Distinct *rpsC* single nucleotide polymorphism lineages of Flavescence dorée subgroup 16SrV-D phytoplasma co-infect *Vitis vinifera* L.. Folia Microbiol..

[41] Mitrovic J., Kakizawa S., Duduk B., Oshima K., Namba S., Bertaccini A. (2011). The *groEL* gene as an additional marker for finer differentiation of ‘*Candidatus* Phytoplasma asteris’-related strains. Ann. Appl. Biol..

[42] Casati P., Jermini M., Quaglino F., Corbani G., Schaerer S., Passera A., Bianco P.A., Rigamonti I.E. (2017). New insights on Flavescence dorée phytoplasma ecology in the vineyard agro-ecosystem in southern Switzerland. Ann. Appl. Biol..

[43] Angelini E., Clair D., Borgo M., Bertaccini A., Boudon-Padieu E. (2001). Flavescence dorée in France and Italy: Occurrence of closely related phytoplasma isolates and their near relationships to palatinate grapevine yellows and an alder yellows phytoplasma. Vitis.

[44] Deng S., Hiruki C. (1991). Genetic relatedness between two nonculturable mycoplasmalike organisms revealed by nucleic acid hybridization and polymerase chain reaction. Phytopathology.

[45] Quaglino F., Zhao Y., Bianco P.A., Wei W., Casati P., Durante G., Davis R.E. (2009). New 16Sr subgroups and distinct single nucleotide polymorphism lineages among grapevine Bois noir phytoplasma populations. Ann. Appl. Biol..

[46] Mitrović J., Siewert C., Duduk B., Hecht J., Mölling K., Broeker F., Beyerlein P., Büttner C., Bertaccini A., Kube M. (2014). Generation and analysis of draft sequences of ‘stolbur’ phytoplasma from multiple displacement amplification templates. J. Mol. Microbiol. Biotechnol..

[47] Hall T.A. (1999). BioEdit: A user-friendly biological sequence alignment editor and analysis program for Windows 95/98/NT. Nucleic Acids Symp. Ser..

[48] Tamura K., Nei M. (1993). Estimation of the number of nucleotide substitutions in the control region of mitochondrial DNA in humans and chimpanzees. Mol. Biol. Evol..

[49] Kumar S., Stecher G., Li M., Knyaz C., Tamura K. (2018). MEGA X: Molecular Evolutionary Genetics Analysis across computing platforms. Mol. Biol. Evol..

