# Supplementary material for: Multilocus Genotyping Reveals New Molecular Markers for Differentiating Distinct Genetic Lineages among “Candidatus Phytoplasma Solani” Strains Associated with Grapevine Bois Noir
Source: Pathogens. 2020 Nov 21;9(11):970. doi: 10.3390/pathogens9110970 (PMC7700334; doi:10.3390/pathogens9110970)

**SUPPLEMENTARY MATERIAL**

**Figure S1**. Unrooted phylogenetic trees constructed based on the nucleotide sequence alignment of the genes *tufB* (a), *glyA* (b), and *tyrS* (c). The highest log likelihood values of trees a, b, c are -3736.99, -3606.24, and -4695.25, respectively. The percentage of trees in which the associated taxa clustered together is shown next to the branches. This analysis involved: 24 sequences with a total of 1185 positions (a); 24 sequences with a total of 933 positions (b); 24 sequences with a total of 1312 positions (c). Strains represent *Ca*Psol lineages identified in this study are in bold; The number (1-15) in parentheses represents *Ca*Psol strain’s lineage; *Ca*Psol strains clustered into *tuf*-type a and b are indicated in blue and black, respectively.


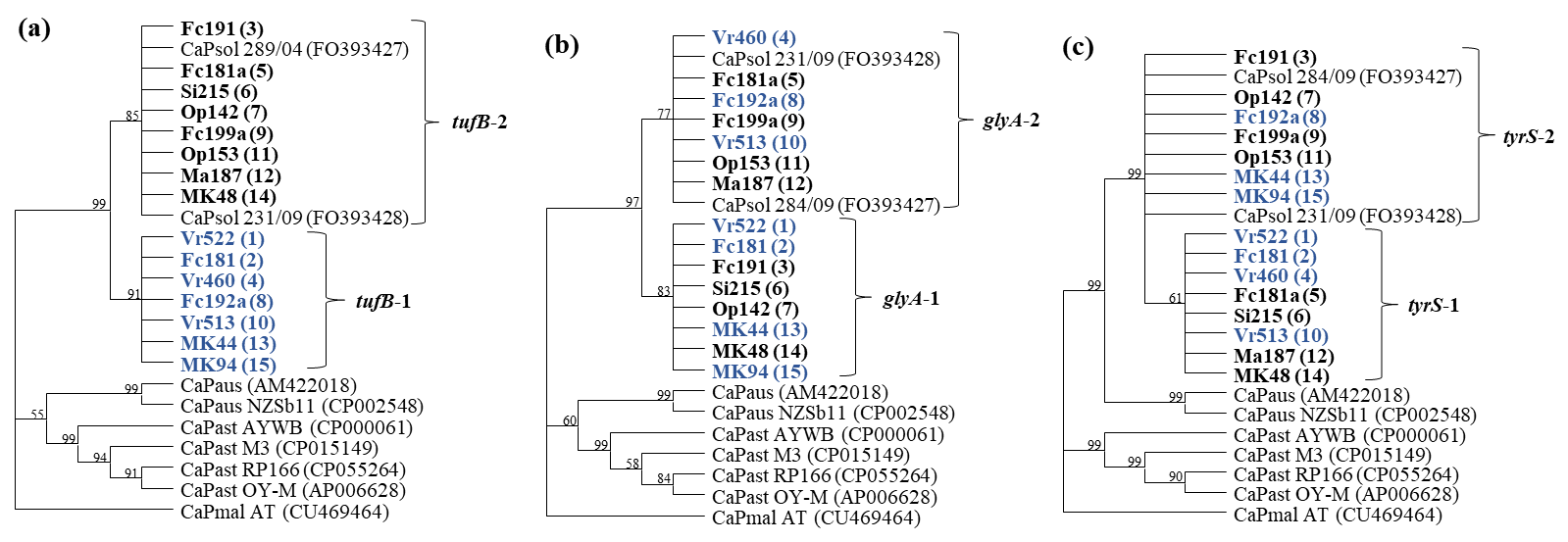

Supplement: Supplementary file 1 [file pathogens-09-00970-s001.zip › SUPPLEMENTARY_MATERIAL_Quaglino_et_al_Pathogens.docx]
